# Leptospirosis is associated with severity, increased resource utilization, and mortality among acute febrile illnesses from Karnataka, Southwestern India

**DOI:** 10.1080/07853890.2026.2687164

**Published:** 2026-06-18

**Authors:** Gautam Raj Panjabi, Gloris Joseph, Kavitha Saravu

**Affiliations:** ^a^ Department of Clinical Immunology and Rheumatology, Medanta Superspecialty Hospital, Indore, Madhya Pradesh, India; ^b^ Department of Infectious Diseases, Kasturba Medical College, Manipal Academy of Higher Education, Manipal, India

**Keywords:** Acute febrile illness, leptospirosis, Sequential Organ Failure Assessment, severity, organ dysfunctions, mortality

## Abstract

**Introduction:**

Acute febrile illness (AFI) is prevalent in tropical regions, with varying etiologies across South Asia. Limited data exists regarding its epidemiology. We aimed to determine the etiology, severity, organ dysfunction, and fatality rate of AFI.

**Materials and Methods:**

This prospective cross-sectional study was conducted at a tertiary care hospital in Southwestern India from September 2016 to August 2018. Hospitalized adults aged >18 years with fever (3–14 days) and no infection foci were included. Patients with co-infections, immunocompromised status, chronic kidney disease, chronic obstructive pulmonary disease, or chronic liver disease were excluded. Severity was assessed using Sequential Organ Failure Assessment (SOFA) score (≥2 indicating severe illness).

**Results:**

Among 314 patients, 66.8% were male. Etiologies included undifferentiated fever (38.5%), dengue (33.1%), scrub typhus (10.2%), leptospirosis (7.6%), malaria (5.1%), and alternate diagnoses (6.4%), with seasonal peaks during monsoon (June to October). Severe illness (SOFA ≥2) was noted in 62.4% of cases. Fatality was 3.2%, with 60% due to undifferentiated fever and 40% due to leptospirosis-associated multi-organ dysfunction.

**Conclusion:**

In this study of acute febrile illness (AFI), at a tertiary care hospital in Manipal, Karnataka, India, etiologies were dengue, scrub typhus, leptospirosis, and malaria. Among these, leptospirosis was associated with the highest median SOFA score, severe hepatorenal and cardiopulmonary dysfunction, required intensive support (inotropes, mechanical ventilation, and hemodialysis), and had the highest fatality rate. Limitations include serological diagnosis of some cases of AFI, lack of convalescent serology in undifferentiated cases, and potential referral bias inherent to a tertiary care hospital.

## 1. Introduction

Fever without localizing signs or symptoms is among the most frequent complaints among persons attending healthcare in tropical countries, particularly during monsoon season. Acute febrile illness (AFI) [1] is defined as a documented temperature of ≥38 °C lasting for 3–14 days without any evident focus of infection. Acute Undifferentiated Febrile Illnesses (AUFI) represent a significant public health challenge in tropical regions, where a wide array of pathogens shares similar clinical presentations. In India, the etiological profile of AUFI is heterogeneous, often influenced by seasonal, geographic, and demographic factors. In India, common etiologies include dengue, enteric fever, scrub typhus, and malaria, which collectively account for a substantial proportion of hospital admissions for acute febrile illness [2]. Recent studies have highlighted dengue as the leading etiology, accounting for 7% to 37%; enteric fever for 8% to 16%; scrub typhus for 14% to 47%; malaria for 6% to 17%; and leptospirosis for 5% to 7% of AUFI cases [3,4]. The incidence of febrile illness may vary from region to region. Very little is known about the epi­demiology of various AFI in different regions of South Asia [5]. Hence, it is very important to determine the epidemiology of the causative pathogens and maintain a comprehensive epidemiological database of various AFIs to establish evidence-based diagnostic algorithms and treatment guidelines. The anticipated diagnostic delay in AUFI, compounded by the limitations of availability of point-of-care testing, often necessitates early empirical intervention. Empiric therapy based on clinical presentation and epidemiological trends is essential to mitigate the risk of rapid progression and severe clinical outcomes [2,6].

In clinical practice, the severity and clinical progression of illnesses are assessed using the Sequential Organ Failure Assessment (SOFA) score. Originally developed as an objective and standardized method to evaluate multi-organ dysfunction in critically ill patients, the SOFA score is widely validated for its prognostic accuracy in infectious diseases. Serial assessment of the SOFA score quantifies the degree of physiological derangement and serves as a reliable indicator of clinical outcome and therapeutic response, facilitating the early identification of high-risk patients who require intensive monitoring and timely intervention [7]. This study aimed to determine the etiological spectrum and clinical characteristics of AFI and to evaluate how specific pathogens and clinical markers assessed *via* SOFA scoring predict disease severity and clinical outcome.

## 2. Methods

### 2.1. Study design, cohort size, patient selection and method

This prospective cross-sectional study was conducted over a period of 2 years, from September 2016 to August 2018, at Kasturba Hospital, Manipal, in the Udupi district of Karnataka State, India. Udupi district is located at 13°32′24.43″ N latitude and 74°52′26.78″ E longitude, with typical tropical climatic conditions. The monsoon period is from June to October, with an average annual rainfall of more than 4000 mm [8]. The catchment area of Kasturba Hospital, Manipal, encompasses both rural and urban populations of coastal and interior Karnataka, Goa, and Kerala. With the expected prevalence of 25% [9] of dengue among AFI, with 5% error and 95% confidence, it was estimated to enrol 288 cases of AFI.

All patients aged ≥18 years admitted to the Kasturba Hospital in a medical unit with acute febrile illness during the study period were included. Patients with evident foci of infection, such as pneumonia, urinary tract infections, meningitis, hepatitis, sinusitis, cellulitis, or those with co-infections or who were immunocompromised with HIV infection, subjects on immunosuppressants, or those with haematological malignancies or autoimmune disorders were excluded. Patients with comorbidities, such as chronic kidney disease, chronic parenchymal liver disease, and chronic lung disease, were excluded.

In all patients fulfilling the inclusion and exclusion criteria, a detailed clinical history was obtained, and a physical examination was performed after written informed consent was obtained. All recruited patients underwent a febrile illness workup as per treating clinicians’ judgement. Diagnostic tests included malaria by Quantitative Buffy Coat (QBC) test and peripheral blood smear; dengue by NS1Ag and IgM ELISA; leptospirosis by IgM ELISA; scrub typhus by IgM ELISA; chikungunya by IgM ELISA; and influenza by throat swab RT-PCR. Blood culture was performed in all patients to rule out bacterial sepsis. Microscopic agglutination test (MAT), polymerase chain reaction (PCR), and culture were not performed routinely in this study. Therefore, leptospirosis cases identified by IgM ELISA using serum samples were classified as probable cases rather than definitively confirmed cases. Other investigations such as complete blood count, renal function tests, serum electrolytes, liver function tests, chest radiography, and urine analysis were also carried out. The severity of the AFI was also evaluated. Organ dysfunction and its severity were ascertained using the Sequential Organ Failure Assessment (SOFA) score. The SOFA score evaluates six organ systems, including respiration, coagulation, liver, cardiovascular, central nervous system, and renal, with each system scored from 0 to 4 points, giving a total score ranging from 0 to 24, with 24 being the worst. The worst SOFA score during the hospital stay was recorded. The patients were categorized according to SOFA score <2 or ≥2. Patients were followed up during hospitalization till discharge, and the clinical outcomes were noted during hospitalization.

### 2.2. Exposure and outcome variables

Demographic, clinical, and laboratory parameters were the exposure variables. The primary outcome was the etiological diagnosis of AFI. Severity assessed by the SOFA score, organ dysfunction, supportive requirements, and case fatality were secondary outcomes. The SOFA Score is a mortality prediction score based on the degree of dysfunction of six organ systems, including the respiratory, cardiovascular, hepatic, coagulation, renal, and neurological systems, with scores ranging from zero to four within each organ system. A SOFA score of ≥2 was considered severe [10]. The outcome was determined in terms of the number of alive/dead patients among all etiologies. Poor outcomes were defined as death or discharge from the hospital in a critical condition.

### 2.3. Statistical analyses

Statistical analyses were performed using SPSS (Statistical Package for the Social Sciences) version 20.0. Descriptive variables were described in terms of counts and percentages for all categorical variables. Continuous variables that were normally distributed were described in terms of mean ± S.D., and continuous variables that were not normally distributed were described in terms of median (IQR). Chi-square test was performed to test the association of categorical variables with different diagnoses. One-way ANOVA was performed to compare the means of continuous variables (normally distributed). The Kruskal-Wallis test was performed to compare the median values of continuous variables (not normally distributed).

## 3. Results

### 3.1. Etiology and demographics

**Figure 1.**
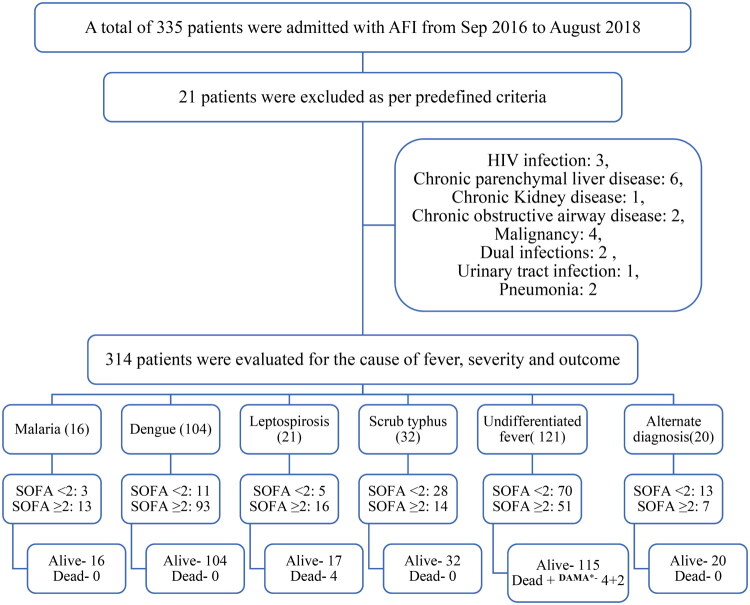
Study flow diagram depicting the etiology, SOFA score and outcomes of adults with acute febrile illnesses (*N* = 314). *DAMA: Discharge against medical advice in critical condition.

**Figure 2.**
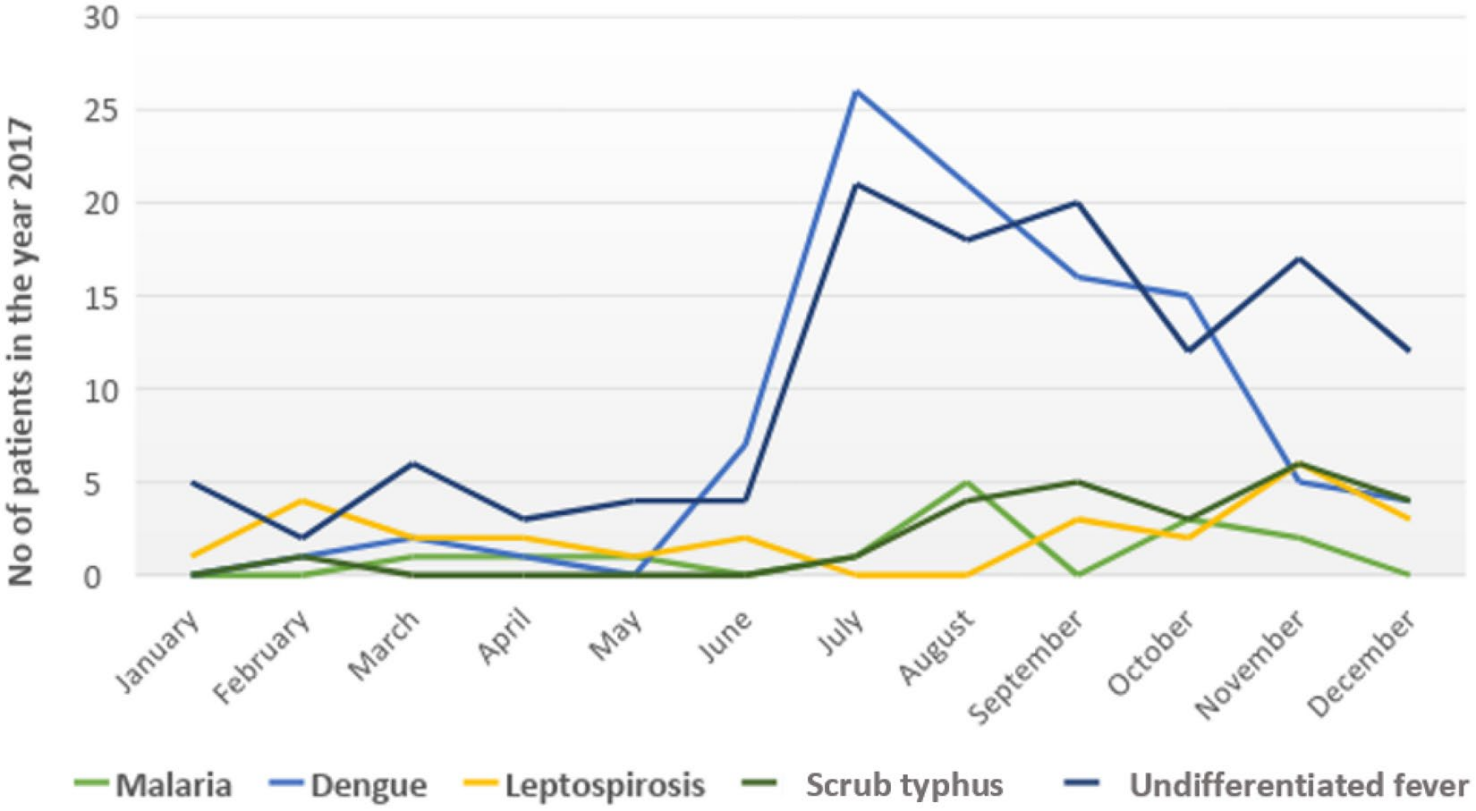
Seasonal trend of acute febrile illnesses observed in one calendar year 2017 from Kasturba Hospital, Karnataka, India.

Among 314 patients enrolled (Figure 1), undifferentiated fever formed the largest group (121 cases, 38.5%), followed by dengue fever (104 cases, 33.1%), scrub typhus (32 cases, 10.2%) (Figure 1). Of all subjects, 210 cases (66.8%) were male; gender distribution varied significantly across AFIs, with male preponderance in patients with malaria and dengue, whereas leptospirosis and scrub typhus showed female preponderance (Table 1). The majority of AFIs occur during the monsoon season (from June to October) (Figure 2).

**Table 1.** Comparison of demographic and clinical variables among adults with acute febrile illnesses.

Variables	Malaria (*N* = 16) n(%)	Dengue (*N* = 104) n(%)	Leptospirosis (*N* = 21) n(%)	Scrub typhus (*N* = 32) n(%)	p-value
Male gender	16(100)	80(76.9)	9(42.9)	15(46)	<0.001
Age, years*	37.4 ± 15.6	38.4 ± 16	37.3 ± 15.3	36.2 ± 14.1	0.868
No of days of fever*	5.8 ± 3.4	5.5 ± 3.1	5.5 ± 2.4	4.8 ± 1.5	0.621
Rash	0(0)	24(30)	2(10.5)	8(33.3)	0.087
Myalgia	5(31.3)	71(68.2)	8(38.1)	16(50)	0.007
Abdominal pain	0(0)	24(23.1)	7(33.3)	7(21.8)	0.028
Breathlessness	0(0)	0(0)	7(33.3)	6(18.6)	<0.001
Icterus	2(12.5)	5(4.8)	9(42.8)	6(18.7)	<0.001
Hepatomegaly	5(31.3)	26(25)	5(23.8)	15(46.8)	0.006
Splenomegaly	8(50)	22(21.2)	0(0)	12(37.5)	0.003
GCS*	14.8 ± 0.5	14.8 ± 1.2	15 ± 0	15 ± 0	0.823
HR, per min*	86.3 ± 13.6	88.3 ± 13.9	78.9 ± 13.5	79.4 ± 9.9	0.011
SBP, mm Hg*	113.7 ± 14.1	120.6 ± 19.6	124.9 ± 17.4	123.3 ± 13.4	0.290
DBP, mm Hg*	73.8 ± 11.5	76.6 ± 11.0	79.0 ± 8.3	79.4 ± 11.3	0.425

* Mean ± Standard Deviation.

GCS: Glasgow Coma Scale; HR: heart rate; SBP: systolic blood pressure; DBP: diastolic blood pressure.

### 3.2. Organ dysfunctions

#### 3.2.1. Respiratory dysfunction

**Figure 3.**
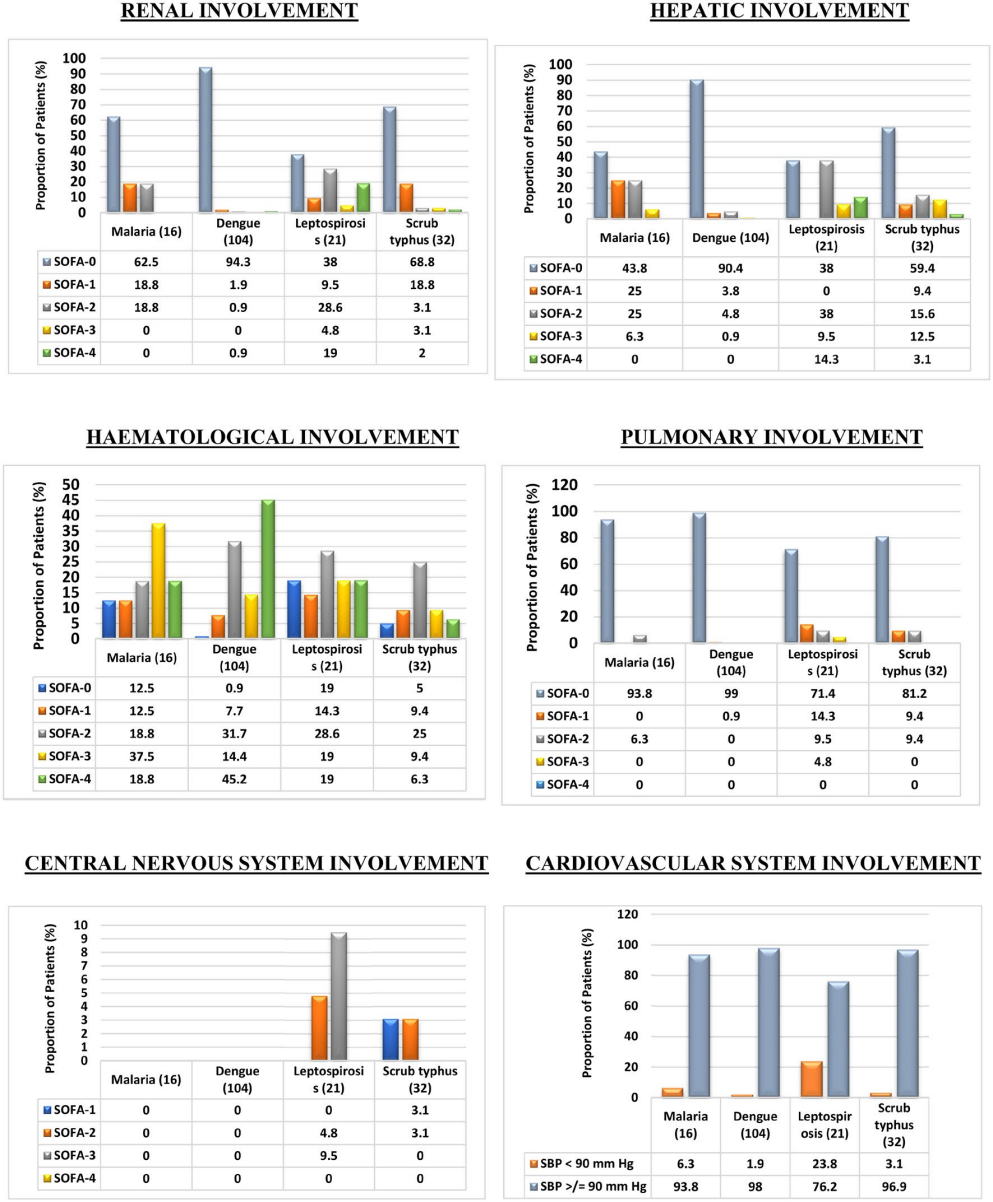
Distribution of various organ dysfunctions among acute febrile illnesses stratified by SOFA score.

**Figure 4.**
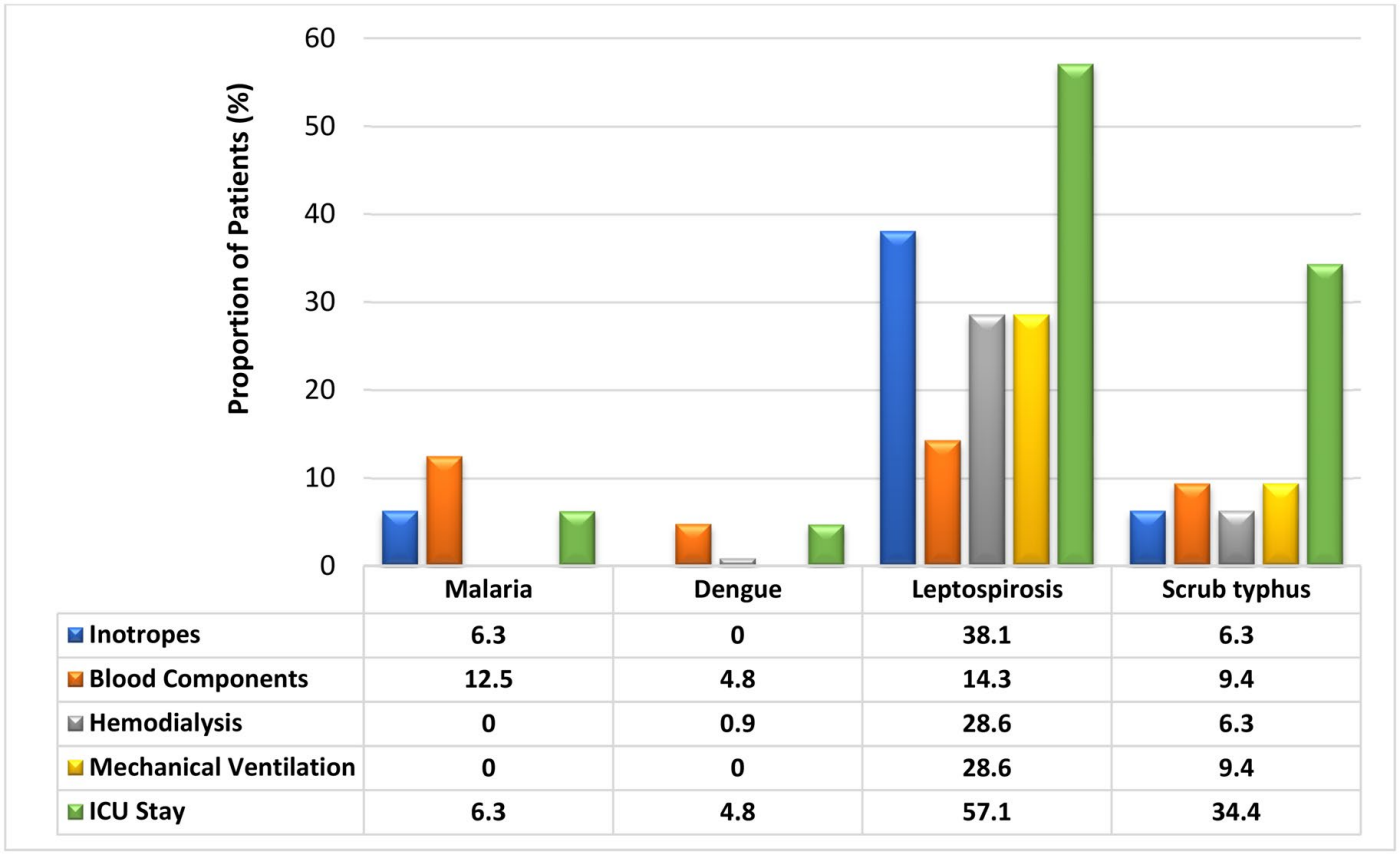
The supportive requirements among adults with AFI’s admitted to Kasturba Hospital. Bar chart comparing five supportive requirements including inotropes, blood components, hemodialysis, mechanical ventilation, and ICU Stay among AFI patients.

Acute respiratory distress syndrome (ARDS) occurred in 14.3%, 9.4% and 6.3% of patients with leptospirosis, scrub typhus, and malaria, respectively (Figure 3). The proportion of patients who were mechanically ventilated (either non-invasive or invasive) was 28.6%, 9.4%, and zero among the three groups, respectively (Figure 4).

#### 3.2.2. Hepatic dysfunction

Bilirubin levels were modestly elevated (2-5.9 mg/dl) in patients with leptospirosis, scrub typhus, and malarial fever with the proportions of 61.9%, 31.3%, and 29.1% respectively, as shown in Figure 3. AST and ALT elevations of approximately 3–5 times the normal range were noted in dengue, leptospirosis, and scrub typhus, whereas in patients with malaria, the hepatic transaminases were in the near normal range. However, an ALP increase of up to three times the normal limit was observed only in scrub typhus, as shown in Table 2.

**Table 2.** Comparison of routine laboratory variables among adults with acute febrile illnesses.

Variables	Malaria (*N* = 16) Median (IQR)	Dengue (*N* = 104) Median (IQR)	Leptospirosis (*N* = 21) Median (IQR)	Scrub typhus (*N* = 32) Median (IQR)	p-value
Hb(g/dl)*	13.3 ± 2.6	12.8 ± 2.1	15.1 ± 1.5	14.8 ± 1.9	0.013
PCV (%)*	40.2 ± 8.2	38.7 ± 6.4	45.3 ± 3.8	43.9 ± 4.5	<0.001
TLC (cells in 100/mm^3^)	53.5(45.5, 63)	44.5(30.5, 68.5)	96(54, 156)	86.5(72.5, 112)	<0.001
Platelets (cells in 1000/mm^3^)	41(25, 103)	31.5(13.0, 67.7)	62(23, 121)	140(67, 214)	<0.001
Total bilirubin (mg/dl)	1.4(0.7, 2.3)	0.6(0.4, 0.9)	2.6(0.5, 6.1)	0.85(0.6, 3.9)	<0.001
AST(IU/L)	27(20, 45)	112(64, 185)	82(39, 220)	84(44, 159)	<0.001
ALT(IU/L)	31(23, 49)	64(42, 116)	66(36, 115)	81(46, 110)	<0.001
ALP(U/L)	85(71, 103)	76(60, 101)	132(62, 192)	193(124, 382)	<0.001
Albumin(g/dl)	3.8(3.3, 4.0)	4.0(3.7, 4.0)	3.1(2.7, 4.0)	2.9(2.6, 3.5)	<0.001
Creatinine (mg/dl)	1.1(1.0, 1.6)	0.9(0.7, 1.0)	2.1(0.9, 3.5)	0.9(0.7, 1.4)	<0.001
Total SOFA score	4(2, 5.7)	3(2, 4)	7(2.5, 10)	1.5(0, 5.7)	<0.001

^*^ Mean ± standard deviation; IQR: interquartile range.

Hb: hemoglobin; PCV: hematocrit; AST: aspartate aminotransferase; ALT: alanine aminotransferase; ALP: alkaline phosphatase; TLC: total leukocyte count.

#### 3.2.3. Renal dysfunction

Acute kidney injury was common in leptospirosis, malaria, and scrub typhus. The proportion of patients who had AKI was 52.4%, 18.8%, and 12.5% of patients, respectively (Figure 3). The proportion of patients requiring hemodialysis at some point in their hospital stay was 28.6%, zero, and 6.3% among the three groups, respectively (Figure 4).

#### 3.2.4. Haematological dysfunction

Leukocytosis (total WBC count >11,000/mm^3^) was noted in scrub typhus and leptospirosis, and normal or low leukocyte counts (total WBC count ≤4,000/mm^3)^ were observed in malaria, dengue, and enteric fever. Thrombocytopenia (total platelet count <150,000/mm^3)^ was commonly observed in patients with dengue (93.4%), malaria (75%), and leptospirosis (66.6%) (Figure 3). Blood products (packed red cells or platelets) were transfused in 14.3%, 12.5%, 9.4%, and 4.8% of patients with leptospirosis, malaria, scrub typhus, and dengue, respectively (Figure 4).

#### 3.2.5. Cardiovascular system dysfunction l

Proportion of patients with hypotension with leptospirosis (23.8%), malaria (6.3%), scrub typhus (3.13%), and dengue (1.9%), as depicted in Figure 3, requiring inotropic support in 38.1%, 6.3%, 6.3%, and none, respectively, at some point of their hospital stay (Figure 4).

#### 3.2.6. Central nervous system dysfunction

Poor sensorium at presentation, as assessed by the Glasgow Coma Scale, was observed in 14.3% and 3.13% of patients with leptospirosis and scrub typhus, respectively, as shown in Figure 3.

#### 3.2.7. Severity proportion and supportive requirement

There were a total of 196 (62.4%) patients who had SOFA scores ≥2 and 118 (37.6%) cases with SOFA scores <2 (Figure 1). As the severity of the SOFA score increased, there was an increase in the requirement of various supports in the form of the need for inotropes, mechanical ventilation, blood components, hemodialysis, and ICU stay due to multi-organ involvement. The proportion of each requirement was higher in patients with leptospirosis and scrub typhus (Figure 4).

#### 3.2.8. Case fatality

Ten cases (3.2%) had a poor outcome, of which 60% were due to undifferentiated febrile illness (6 cases) and 40% due to leptospirosis (4 cases), and all of which were due to multi-organ failure. Mortality in patients diagnosed with leptospirosis was 19% in our study. No mortality was observed among patients diagnosed with other tropical illnesses in our cohort.

#### 3.2.9. Treatment details

Management of patients was dictated by clinical judgement of the attending physicians, which includes crystalline penicillin/ceftriaxone/doxycycline for leptospirosis; doxycycline or azithromycin for scrub typhus; chloroquine followed by primaquine for *Plasmodium vivax* malaria; and artemisinin combination therapy for *Plasmodium falciparum* malaria. All patients had received supportive care. Information regarding the administration of appropriate antibiotics was recorded for all study participants. In the cohort, appropriate antibiotics were received by 16 (100%) malaria patients, 20 (95.2%) leptospirosis patients, and 31 (96.9%) scrub typhus patients.

## 4. Discussion

### 4.1. The disease burden of acute febrile illnesses

A major group of AFI patients had undifferentiated AFIs, constituting 38.5% of all cases, a similar observation was noted in the studies conducted in the rural part of Kerala [11] and Thailand [12]. In a study from Mangalore (adjacent district to the study location), the overall leptospirosis frequency rate was found to be 21%, which is consistent with the recognized endemicity of leptospirosis in coastal Karnataka and its seasonal rise during the monsoon months [13]. Dengue fever was noted to be the most common identifiable cause of AFI in the current study, constituting one-third (33.1%) of all cases, and was also the leading cause in one of the hospital-based studies conducted in Mumbai (25%) [9]. However, a study conducted at Vellore [14] noted scrub typhus to be the most common etiology, constituting approximately 47.5% of all AFI, whereas our study noted 10.2% of the cases of AFI being diagnosed with scrub typhus, as depicted in Figure 1. This highlights the heterogeneity of major AFI’s across different geographical regions in India.

### 4.2. Gender and seasonality

Male preponderance in malaria and dengue is attributed to gender-specific work patterns, as males are usually engaged in outdoor activities and practise sleeping outdoors, predisposing them to an increased risk of mosquito bites (Table 1). Similar observations were noted in the studies by Manipal [15] and Mumbai [9]. Female preponderance in scrub typhus and leptospirosis individuals could probably be because women often look after agricultural work, comparable to a study from Northern India [16] which also noted similar findings. The majority of these illnesses occurred during the monsoon season from June to October of the calendar year 2017 (Figure 2), as reported in other studies [14,16].

### 4.3. Severity proportion and organ dysfunctions among AFI’s

Patients who had SOFA ≥2 (severe illness) constituted 62.4% of all the hospitalized AFI cases, probably because the majority of our patients presented late in the course of illness after being complicated with multi-organ failures, and those with subclinical or mild self-limiting illness didn’t report to the hospital or were treated as ambulatory cases. Patients with leptospirosis and scrub typhus fever were more morbid, as stratified by SOFA, and required multiple supportive measures as a consequence of multi-organ involvement (Figure 4).

#### 4.3.1. Respiratory disease

Pulmonary involvement in leptospirosis patients varies from 20% to 70%, depending on the severity of illness [17]. The proportion of ARDS in scrub typhus in different hospital-based studies conducted in Vellore [14] and northeastern Thailand [12] was 24.9% and 5.4%, respectively. ARDS in patients with severe *Plasmodium falciparum* is reported in 5% to 25% of adults [18]. In a study from Southwestern India [15], the proportion of ARDS in patients with *Plasmodium vivax* was 9.8%. The pathogenesis of lung injury/ARDS involves the marked production of inducible nitric oxide synthase (iNOS) and nitric oxide [19]. The pathogenesis of ARDS in malaria is linked to sequestration of infected erythrocytes in the lungs and/or immune-mediated increased leakage from the pulmonary vessels, leading to diffuse alveolar damage [18]. Direct endothelial cell invasion and marked inducible nitric oxide synthase expression may be involved in the pathogenesis of ARDS associated with scrub typhus [20]. ARDS in our study occurred in 14.3%, 9.4%, and 6.3% of patients with leptospirosis, scrub typhus, and malaria, respectively (Figure 3), with ventilatory requirements in 28.6% and 9.4% of individuals with leptospirosis and scrub typhus, respectively, and none in malaria cases.

#### 4.3.2. Hepatic involvement

Bilirubin levels were modestly elevated (2-5.9 mg/dl) in patients with leptospirosis, scrub typhus, and malarial fever, with proportions of 61.9%, 31.3%, and 29.1%, respectively. AST and ALT elevations of approximately 3–5 times the normal range were noted in dengue, leptospirosis, and scrub typhus, whereas in patients with malaria, the hepatic transaminases were in the near-normal range. This aligns with a Vellore study where mild elevations (3–5 times the upper limit of normal) in AST and ALT were observed in 70% of patients with scrub typhus [14]. In the current study, an ALP increase of up to three times the normal limit was observed in scrub typhus only, which could be a clue that guides its diagnosis among AFI’s.

*Orientia tsutsugamushi* causes liver dysfunction by triggering focal inflammation due to intrahepatic sinusoidal vasculitis and direct cytopathic hepatic injury [21]. Jaundice in leptospirosis is possibly due to hepatocellular necrosis, intrahepatic cholestasis, and increased bilirubin load following the absorption of tissue hemorrhage [22]. Hepatic dysfunction in malaria is multifactorial, and different factors include intravascular red cell lysis, glucose-6-phosphate dehydrogenase deficiency-related hemolysis, antimalarial treatment, or sepsis-induced liver dysfunction [23].

#### 4.3.3. Renal involvement

The proportion of AKI in leptospirosis patients is 52.4% in the current study, highlighting the profound renal dysfunction associated with leptospirosis. In a hospital-based study by Vellore [14], the proportion was 16.7%. Possible mechanisms causing renal injury are hypotension, immune-mediated injury, and direct nephrotoxicity to the co-transporter in the renal tubules. Acute tubular necrosis (ATN) and interstitial nephritis (AIN) occur in patients with leptospirosis [24]. Renal impairment occurred in 18.8% of cases with severe falciparum malaria in our study, whereas 40% reported in a study from Vellore [14]. Probable mechanisms linked to its occurrence are erythrocyte sequestration impeding renal microcirculation, hypovolemia, and hemolysis-induced renal injury [25]. The proportion of AKI in scrub typhus in the current study was 12.5%, whereas in a study from Northern India [25], scrub typhus was complicated by acute renal failure in 22.9% of patients. Possible mechanisms that could contribute to renal injury in scrub typhus include hypotension and acute tubular necrosis as a result of direct invasion by bacteria. The proportion of patients with AKI requiring hemodialysis in the present study cohort was 28.6%, 6.3%, and none in the leptospirosis, scrub typhus, and malaria groups, respectively (Figure 4).

#### 4.3.4. Haematological involvement

Leucocytosis was noted in scrub typhus and leptospirosis; normal leukocyte counts or leukopenia was noted in the majority of malaria, dengue, and enteric fevers. In a study from Manipal, India, leukopenia was observed in 24.3% and 22.4% of the falciparum and vivax cohorts, respectively [26]. Thrombocytopenia was commonly observed in the patients with malaria, dengue, and leptospirosis (Table 2). About 86.4% of *P. falciparum* patients and 90.5% of *P. vivax* patients had thrombocytopenia in a study from Manipal [26].

Possible mechanisms postulated for thrombocytopenia in malaria include immune-mediated thrombocytopenia, splenic sequestration, platelet activation and aggregation [26]. Thrombocytopenia in dengue could be due to platelet destruction secondary to immune complex formation, complement activation, and bone marrow suppression [14].

#### 4.3.5. Cardiovascular system involvement

Hypotension was seen more commonly in patients with leptospirosis (23.8%), malaria (6.3%), scrub typhus (3.13%), and dengue (1.9%), requiring inotropic support in 38.1%, 6.3%, 6.3%, and none, respectively (Figure 4). Possible mechanisms that could have contributed to hypotension in our patients included sepsis syndrome and myocarditis. In patients with dengue, other probable causes include persistent vomiting, extensive capillary leakage, and extensive bleeding, resulting in hypovolemia.

#### 4.3.6. Central nervous system involvement

In a study conducted at Vellore [14], altered sensorium had mainly occurred in patients with scrub typhus (53.6%) and falciparum malaria (18.8%). Altered sensorium is caused by aseptic meningitis, encephalitis, or hypotension. Poor Glasgow Coma Scale scores were noted in our study in 14.3% and 3.13% patients with leptospirosis and scrub typhus, respectively, as depicted in Figure 3.

#### 4.3.7. Case fatality

Case fatality rate in our study was 3.2% (10 cases), out of which 60% were due to undifferentiated febrile illness and 40% due to leptospirosis, all of which were due to multi-organ failure. In a hospital-based study from Kerala [11], India, a case fatality of 1.7% was noted among AFIs and of all, leptospirosis was the foremost etiology (85% of the deaths). Thus, among AFIs, leptospirosis contributes significantly to the morbidity characterized by hypotension, hepatorenal, and pulmonary dysfunction requiring maximum support, such as inotropes, mechanical ventilation, and hemodialysis, and can be fatal. Mortality in patients diagnosed with leptospirosis was 19% in our study, which is higher than the overall case fatality of approximately 5–6% reported in global burden analyses [27]. The higher mortality observed in our study may reflect the tertiary-care hospitalized nature of the cohort, referral of more severe cases, delayed presentation, and differences in diagnostic criteria. In studies done previously, the fatality rate in patients admitted with leptospirosis ranged from 4% to 52% [28]. Therefore, early identification by clinico-laboratory correlations and aggressive management is essential to prevent death. No mortality was observed in the patients diagnosed with other tropical illnesses in our study.

## 5. Strengths and limitations

The SOFA-based assessment for organ dysfunction was described, which is a widely accepted, simple, and easily calculated tool to assess the severity of patients. Severity based on standard scoring will allow for easy comparison in future studies.

All SOFA variables were not available, as they were not deemed necessary by treating physicians for case management; for example, arterial blood gas analysis was unavailable for stable patients, especially in medical wards with normal oxygen saturation. In such patients, the SOFA score for pulmonary involvement was determined to be zero. Molecular assays (e.g., PCR) and gold-standard serology (e.g., MAT for leptospirosis) were unavailable in routine care; stronger IgM responses in severe disease may have caused cross-reactivity and false positives. Undiagnosed cases after routine workup were thus classified as undifferentiated febrile illness, with potential misclassification bias from lacking advanced diagnostics. We did not perform diagnostic convalescent serology for patients with undifferentiated fever, which could have helped to reclassify some cases from undifferentiated to specific etiologies. The higher severe AFI proportion likely reflects the hospital’s referral nature. Treatment followed routine clinical practice as per clinicians’ discretion. Follow-up was restricted to short-term outcomes until hospital discharge, precluding assessment of long-term sequelae such as persistent organ dysfunction. Future research with more standardized treatment protocols and comprehensive data capture is necessary to better characterize the independent impact of etiology on clinical outcomes.

## 6. Conclusions

In this study conducted at a tertiary care hospital in Manipal, Karnataka, India, etiology of AFI remained unidentified in 38.5%, with dengue, scrub typhus, and leptospirosis being most common among diagnosed cases. AFI cases showed seasonal preponderance during monsoon (June-October); with SOFA scores ≥2 in 62.4% and case fatality in 3.2% of cases. Leptospirosis and scrub typhus cases were associated with leukocytosis and hepatorenal/cardiopulmonary involvement; leptospirosis was associated with higher severity, increased resource utilization, and mortality among acute febrile illnesses. These findings, limited by referral bias, lack of confirmatory diagnostics in leptospirosis and scrub typhus, and short-term follow-up, highlight the need for enhanced molecular testing to clarify etiology, clinical severity and outcomes of AFI in tropical settings.

## Data Availability

The data will be made available on reasonable request to the corresponding author (KS).
